# P07 Antimicrobial stewardship in Ireland 2009–22: a scoping review

**DOI:** 10.1093/jacamr/dlad143.011

**Published:** 2024-01-03

**Authors:** Fiona Barry, Maura Smiddy, Tony Fitzgerald, Eilis J O’Reilly, Olive Murphy

**Affiliations:** School of Public Health, University College Cork, Cork, Ireland; School of Public Health, University College Cork, Cork, Ireland; School of Mathematical Sciences and School of Public Health, University College Cork, Cork, Ireland; School of Public Health, University College Cork, Cork, Ireland; Bons Secours Hospital, Cork, Ireland

## Abstract

**Objectives:**

The aim of this scoping review is to examine the current literature to evaluate progress related to the implementation of the Strategy for the Control of Antimicrobial Resistance in Ireland (SARI) Hospital Antimicrobial Stewardship (AMS) Working Group guidance (2009) on antimicrobial stewardship in hospitals in Ireland.

**Methods:**

Systematic searches were carried out on four electronic databases (PubMed, CINAHL, Embase, Web of Science) and five targeted websites for grey literature sources. Titles and abstracts were screened independently by two reviewers. The remaining full-text publications were reviewed by all authors. Studies were mapped into at least one of four categories defined by recommendation headings from the SARI Hospital Antimicrobial Stewardship Working Group guidance: (A) structure and organization of AMS; (B) roles and responsibilities for prescribers; (C) antimicrobial stewardship interventions; (D) recommendations for non-acute residential healthcare institutions.

**Results:**

The search yielded 545 peer reviewed papers for screening of which 35 were included and 327 grey reports of which 26 were included. Most articles were mapped to (C) antimicrobial stewardship interventions, with the least number mapped to (A) structure and organization of AMS. The most consistent findings in (C) were numerous implementation challenges requiring commitment by hospital leadership to provide human and financial resources, need for improved diagnostic capacity, and educational pathways for prescribers, pharmacists, nurses and patients. The findings in (A) included inadequate staffing across hospitals to implement stewardship programmes (regardless of hospital acuity) and investment required for information and communication technology resources to further support AMS and infection prevention and control.

**Conclusions:**

There has been progress on AMS over the last 20 years and implementation remains a significant challenge. Clear identification of AMS programme management, use of data, education and communication need to be considered for the success of future implementation in all healthcare settings.

